# A critical review of anthropological studies on skeletons from European plague pits of different epochs

**DOI:** 10.1038/s41598-018-36201-w

**Published:** 2018-12-05

**Authors:** B. Bramanti, N. Zedda, N. Rinaldo, E. Gualdi-Russo

**Affiliations:** 1 Department of Biomedical and Specialty Surgical Sciences, University of Ferrara, Ferrara, Italy; 2 Centre for Ecological and Evolutionary Synthesis, Department of Biosciences, University of Oslo, Oslo, Norway; 3 University Center for Studies on Gender Medicine, University of Ferrara, Ferrara, Italy

## Abstract

In historical times, plague epidemics intermittently ravaged Europe for more than 1,400 years, and still represent a threat in many countries all over the world. A debate is ongoing about the past plague, if it killed randomly in a population or discriminated among persons on the basis of their biological features. To address questions of plague lethality, we reviewed a large number of anthropological studies published in the last twenty years on victims of the past pestilences in Europe. In particular, we focused on data concerning demography (age at death and sex determination), and health status (skeletal biomarkers). We applied to these data a model system based on Multiple Linear Regression, which aimed to discern among possible predictors of sex-selective plague lethality in entire populations, in different periods and regions. Based on available data, we lack evidence for general trends of association between biological features. Differences in sex ratio are more likely due to the original population compositions or to distinct cultural behaviours of the two genders. We concluded that generalizations on biological evidence are not feasible for ancient plagues if we exclude that the infection possibly killed primarily persons between 5–10 and 20–35 years of age.

## Introduction

Plague is an infectious disease that has repeatedly caused small and large epidemics throughout history. From historical and artistic works, we know that, since the sixth century, Europe has experienced epidemics of plague. The first recorded plague pandemic started during the Justinian empire in 541 CE in Egypt and spread rapidly throughout the Mediterranean Basin and the northern part of Europe, up to the Rhine region and to Ireland^1^. The outbreaks of this period, recorded until 750 CE, are now referred to as the First Pandemic since the disease was prevalent on at least two continents (Europe and Africa). The Second Pandemic started in Asia and spread to the whole of Europe in 1347 CE from Kaffa on the Black Sea. The first period of this pandemic is called the Black Death (1346–1353), after which plague persisted in Europe until the beginning of the nineteen century (Malta 1813^2^). Shortly before (1772), a major epidemic began in the Yunnan Province of Southwest China^3^. The disease spread to Hong Kong in 1894 and from there it was disseminated globally via maritime shipping, giving rise to the Third Pandemic^4,5^.

In the past, particularly in medieval and early modern times, plague spread rapidly to European regions connected by trade^6,7^ and produced a large number of deaths. Throughout the acutest phase of an epidemic, the victims were buried in multiple graves or large pits. Nevertheless, mostly at the beginning or at the end of an outbreak, the victims were also buried singularly in regular cemeteries or in multiple burials with few victims each^8^.

Archaeologists have recovered many pits and multiple graves in different regions of Europe and beyond, both in rural and urban contexts, which have been attributed to past plague pandemics. Bioarchaeologists have often carried out anthropological studies on those skeletons, collecting biological data that have been used to evaluate the demographic composition (sex and age at death) as well as indicators of skeletal stress (body dimensions and paleopathological lesions). Information on skeletal stress is essential to assess relationships with health status, morbidity and mortality^9,10^. Often, bioarchaeologists have aimed to address the question if plague was a “universal killer” or if it was selective in its mortality effects, with regard to the pre-existing health status of the victims determined on skeletal indicators, to their sex or age. The majority of the studies concluded that mortality due to plague was not selective in respect to sex (*e*.*g*.^11–14^), age (*e*.*g*.^12,15^), and likely health status^16,17^. Historical, paleodemographic and paleoepidemiological studies support as well the view of plague killing indiscriminately, but only during the largest outbreaks, the Black Death^18,19^ and the outbreaks of the 17^th^ c. in Italy^20^. Outbreaks of the 14^th^ c. successive to the Black Death were often referred to by chronicles as *pestis puerorum*, since they killed prevalently children. This view is now confirmed by paleodemographic studies^21–23^, yet the agreement is not complete^24^. Finally, from the 15^th^ c. onwards, plague was proposed to have assumed a “social character” mostly striking the poorest classes^24–26^.

Recently, some scholars working on bioarchaeological data from London have challenged the view of the Black Death killing indiscriminately and have identified elements of selectivity for age and pre-existing health status of the victims^27–30^. Another anthropological study^31^ has demonstrated that the Black Death killed in England more male than female individuals among those who showed more indicators of skeletal frailty due to pre-existing physiological stress. The outcomes of this study can also be explained by a lower risk of death due to plague for healthy men than healthy women. An additional study, carried out on mortmain records in Hainaut, Belgium, in the period 1349–1450^32^ has revealed that the Black Death (which hit in the region 1349–51), as well as other plague epidemics up to 1450, often had a sex-selective effect and killed more women than in “non-plague years”. The authors propose that a biological reason might explain their outcomes more than a cultural one, which could have enhanced the risk of exposure to plague for women.

The majority of the conclusions of bioarchaeological studies were drawn from single plague pits or cemeteries of different periods, often compared with a reference (i.e. “non-plague”) population. Few exceptions are represented by works comparing 2–7 burial grounds^11,14,17,33,34^.

Here, we propose to describe the largest number of archaeological sites, which have been attributed to plague on the basis of molecular tests or of relevant historical records, and for which data are published, and to test on these data some of the hypotheses of selective plague mortality. The goal of this review is to compare different studies on European plague burials to observe if previous conclusions can be extended to all outbreaks and a homogeneous trend for plague mortality on the basis of biological features is recognizable. In particular, we aimed to test whether differentiated sex patterns can be associated with markers of frailty in populations of plague victims from distinct European regions and different plague outbreaks.

We proceed therefore in reviewing a large number of bioarchaeological studies carried out on plague victims of the past. These remarkable works are often relegated to local publications in different languages and are frequently not easy to find in a digitized version. Although limited to publications in English, French, Italian and German, this investigation represents the largest review ever published on this topic. Previous works include a critical discussion on the attribution to plague of several German mass graves in the pre-genomic era^35^; a catalogue of mass graves – or possible mass burials – of the period around the Justinian plague^36,37^ and a general survey of some published literature on later medieval cemeteries^38^.

For the comparison among different archaeological sites, we considered different possibilities. One of the most used methods for evaluating plague mortality is the hazard model^27,28^, which allows us to relate skeletal lesions with higher risk of mortality at the individual level. A recent study^10^ has proposed to calculate for each individual an index of skeletal frailty (SFI), which can be stratified by sex and age and applied to the broader population level. Yet, the published works here reviewed do not generally include data at the individual level (with the remarkable exceptions of the East Smithfield cemetery of London (1348–1349), from the open-access MoL Wellcome Osteological Research Database, WORD: http://archive.museumoflondon.org.uk/Centre-for-Human-Bioarchaeology/Database/ and the PhD thesis of S. Kacki 2016^17^). Thus, the use of these statistical analyses remains beyond the possibilities of the current study.

As an alternative, we resort to a statistical descriptive approach to appreciate homogeneities and differences across the archaeological sites for the biomarkers under observation. Further, we attempted to address predictors of a possible sex-based lethality using Multiple Linear Regression Models.

## Methods

**Figure 1:**
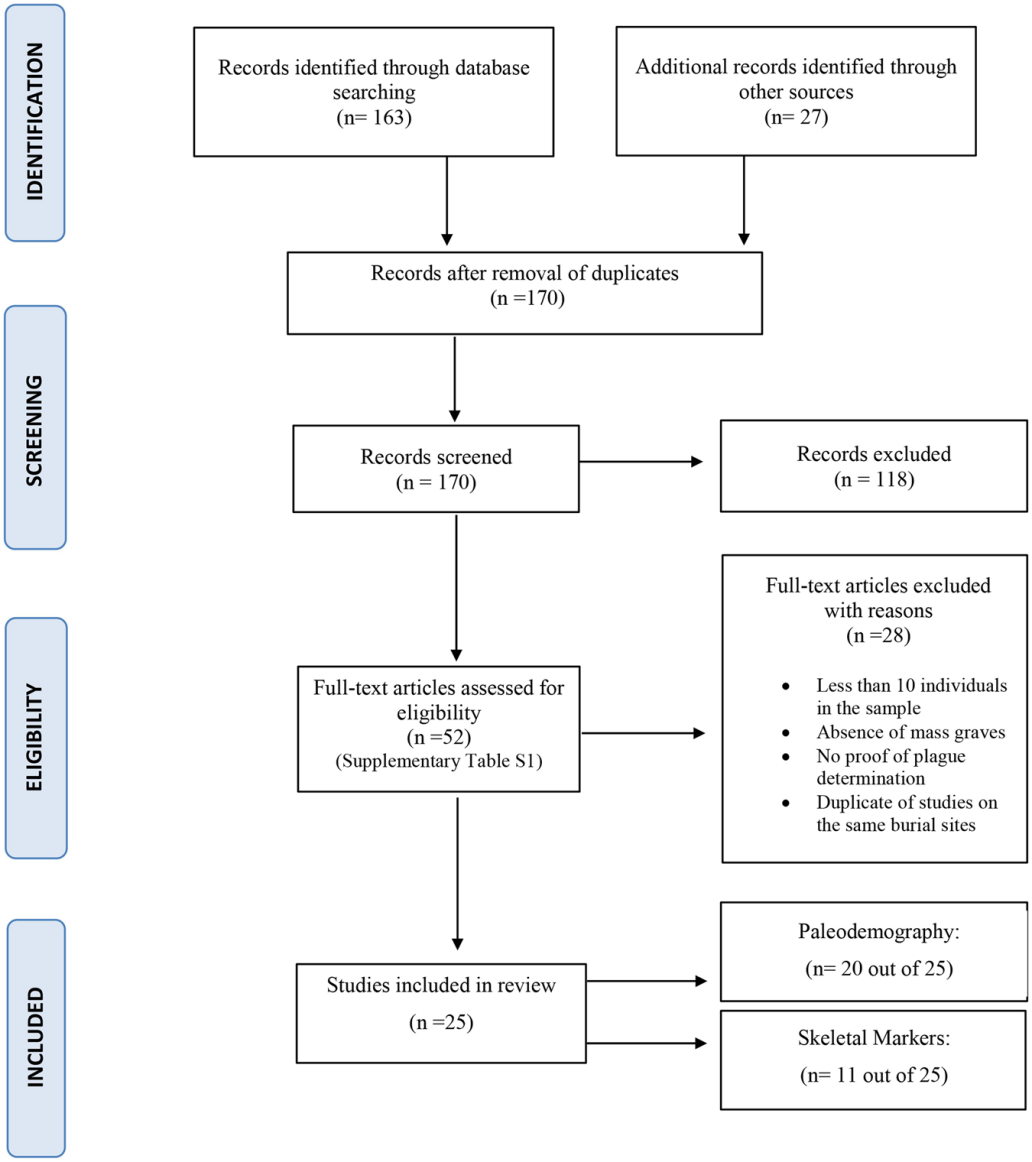
PRISMA Flowchart^39^.

In December 2016, we systematically searched for articles describing anthropological studies on victims in PubMed and BioMedSearch databases. We limited our search to papers published during the last 20 years (1996–2016) and, initially, only to publications in English (see Fig. 1). For our research, we used the following terms: ‘plague’, ‘*Yersinia pestis’*, ‘Black Death’, ‘Justinian plague’ combined in turn with ‘skeleton’, ‘bones’, ‘skeletal markers’, ‘paleopathology’, ‘paleodemography’, ‘demography’, ‘age’, ‘sex’. Moreover, we carried out a direct search for articles published in the three following anthropological journals: American Journal of Physical Anthropology, Homo, and International Journal of Osteoarchaeology. Subsequently, given the limited number of articles found, we extended our search to the reference lists of the retrieved studies, which included works published in German, Italian and French, and to other informative websites. We checked the texts for relevance according to pre-determined inclusion criteria (studies with age and sex determination and/or examination of pathological lesions, carried out on skeletons). Finally, we considered some published and digitalized books and PhD theses that contributed significantly with information not provided by other publications on the same archaeological sites. The 52 publications selected under these criteria are listed in the Electronic Supplementary Material (Supplementary Table S1).

Further criteria limited the eligibility of the publications for statistical analyses to only 25 studies. Limiting criteria were: 1) the presence of at least ten individuals in the examined samples was considered mandatory to generate frequencies of biological markers; 2) skeletons should come from multiple burials or from mass graves, or also from single graves if they belong to the same burial ground and are contemporary to the multiple or mass graves (as attested by historical or archaeological evidence), for which 3) the death of the victims should be due to plague, as stated by historical and/or paleomolecular and/or paleoimmunological evidence, according to the same article or other ones from the literature (sources of information are given in Supplementary Table S1). When multiple studies on the same burial site were found, we used the report presenting numerical data rather than graphs for demographic analysis. Further, we selected the study with the largest sample size and, as the next criterion, the most recent and informative one.

From the 25 studies here considered, we obtained useful data relative to 23 plague burials, 4 sites of the First Pandemic and 19 sites of the Second Pandemic (Table 1), which were used for statistical purposes, to evaluate anthropological differences and similarities among distinct plague sites concerning sex distribution, age at death and stress markers. Differences in age at death and sex distribution (the latter determined only in adults) were inferred using a χ^2^-test. The results of the χ^2^-tests were considered significant when the *p*-value was ≤0·05. With regard to the age, we would have preferred to consider a distribution in different age classes. Unfortunately, each study used different criteria of age classification, so that the only possible statistical comparison was between sub-adults (aged 0–19 years) and adults (from 20 years on). This is a general and obvious limitation of reviewing studies. Nevertheless, we tried to avert this constraint by reporting and representing graphically the most frequent age classes retrieved in each archaeological site.

**Table 1:** Studies on skeletal remains of plague victims: sex and age at death.

Geographic area	Site	Period	Sample n.	M n. (%)	F n. (%)	sex ND n. (%)	Sub-Adults n. (%)	Adults n. (%)	Age ND n. (%)	Age class most represented		Ref.
										Age class	n. (%)	
Southern Germany	Aschheim	5^th^–7^th^ cent.	77	27 (35%)	40 (52%)	10 (13%)	26 (34%)	50 (65%)	1 (1%)	20–40 40–60	23 (30.3%) 21 (27.4%)	^77^
Southern Germany	Altenerding	5^th^–7^th^ cent.	20	5 (25%)	6 (30%)	9 (45%)	9 (45%)	11 (55%)		20–40 0–6 7–12	9 (45%) 4 (20%) 4 (20%)	^78^
Northern France	Le Clos des Cordeliers, Sens, Yonne	5^th^–6^th^ cent.	73	14 (31.11%)	17 (37.8%)	14 (31.11%)	28 (38%)	45 (62%)				^14^
Southern France	Place Camille Jouffray, Vienne, Isère	760–880	11	1 (20%)	3 (60%)	1 (20%)	6 (55%)	5 (45%)		15–19 40+	3 (27.3%) 3 (27.3%)	^15^
**Second Pandemic: 14**^**th**^**–18**^**th**^ **cent**.												
UK	Royal Mint, East Smithfield, London	1348–1349	600	210 (50%)	167 (39%)	46 (11%)	177 (29.5)	421(70%)	2 (0.5%)	5–15 25–35	109 (18%) 99 (16.5%)	^79^
UK	Hereford cathedral	14^th^ cent.	185	40 (38%)	52 (49%)	14 (13%)	79 (43%)	106 (57%)		5–9 20–39	28 (15%) 25 (13.5%)	^17^
Southern Germany	Manching Pichl, Ingolstadt	1250–1500	21	13 (62%)	6 (29%)	2 (9%)	12 (57%)	9 (43%)		20–40 0–6	6 (28.6%) 5 (23.8%)	^80^
Northern France	Saint-Pierre de Dreux (Eure-et-Loire)	14^th^ cent.	69	11 (34%)	15 (47%)	6 (19%)	37 (54%)	32 (46%)		20+ 1–4	32 (46.3%) 15 (21.7%)	^17^
Northern France	Bondy	1297–1373	12	3 (43%)	1 (14%)	3 (43%)	5 (42%)	7 (58%)				^81^
Southern France	Vilarnau, Roussilon	14^th^ cent.	19	3 (37.5%)	5 (62.5%)		11 (58%)	8 (42%)				^82^
Southern France	Saint Come et Damien, Montpellier	14^th^ cent.	13	1 (17%)	3 (50%)	2 (33%)	7 (54%)	6 (46%)				^83^
Southern France	Saint-Laurent-de-la-Cabrerisse	14^th^ cent.	13	6 (60%)	3 (30%)	1 (10%)	3 (23%)	10 (77%)		20–49 50+	5 (31%) 2 (15%)	^84^
Northern Italy	Lazzaretto Vecchio, Venice	14^th^–17^th^ cent.	331				92 (28%)	192 (58%)	47 (14%)			^85^
Spain	Basilica dels Sants Just i Pastor, Barcelona	14^th^ cent.	120	7 (10%)	6 (9%)	57 (81%)	50 (42%)	70 (58%)		5–9 20+	13 (11%) 70 (58%)	^16^
Belgium	Maria Troon, Dendermonde	16^th^ cent.	99	20 (45%)	18 (41%)	6 (14%)	55 (56%)	44 (44%)		5–9 10–14	22 (22.2%) 17 (17.2%)	^17^
Southern France	Les Fedons, Lambesc	16^th^ cent.	133	30 (50%)	26 (43%)	4 (7%)	73 (55%)	60 (45%)		5–9 20–30	24 (18%) 17 (12.8%)	^17^
Southern Italy	Lo Quarter, Alghero	1582–1583	185	43 (45%)	51 (53%)	2 (2%)	89 (48%)	96 (52%)		12–20 3–11	38 (20.5%) 36 (19.5%)	^86^
Southern France	Puy St. Pierre, Lariey	1629–1630	34	8 (47%)	8 (47%)	1 (6%)	17 (50%)	17 (50%)		40–60 5–9	8 (23.5%) 7 (20.6%)	^87^
Northern Italy	Osservanza, Imola	1629–1630	92	33 (36%)	39 (42%)	20 (22%)	43 (47%)	49 (53%)		20–35 12–20	27 (29%) 20 (22%)	^88^
Denmark	Copenhagen	1711–1712	24				8 (33%)	16 (67%)				^89^
Southern France	l’Observance, Marseille	1720–1722	179	59 (46%)	58 (45%)	11 (9%)	51 (28.5)	128 (71.5%)		5–9 20+	20 (11.2%) 128 (71.5%)	^90^
Southern France	Le Delos, Martigues	1720–1722	39	9 (41%)	5 (23%)	8 (36%)	17 (44%)	22 (56%)		0–5 20–35	6 (15.4%) 6 (15.4%)	^91^
Southern France	Le Couvent des Capucins de Ferrieres, Martigues	1720–1722	208	56 (46%)	52 (43%)	14 (11%)	86 (41%)	122 (59%)				^92^

ND: undetermined. Sex determination is relative to adults only, but for the samples from Aschheim, Manching Pichl, and Osservanza (Imola), for which also subadults were considered.

This review was carried out in accordance with the PRISMA guidelines^39^.

### Predictive models

Further, we attempted to identify predictors for the differences in sex-ratio (*N*_m_ /*N*_f_) among the indicators of health status. Only 7 sites could be considered, for which data on skeletal markers of biological stress (at least Hypoplasia, Cribra Orbitalia, Porotic Hyperostosis) were present (Table 2 and Supplementary Table S2). All these plague pits were attributed to the Second Pandemic: Saint-Pierre de Dreux, Barcelona, Royal Mint, Hereford, Les Fedons, Maria Troon and Puy St. Pierre. Since sex determination cannot be attested with confidence in subadults, in each population and for each variable, we used frequencies only from adult individuals (Table 2 and Supplementary Table S2).

**Table 2:** Frequency of biomarkers of stress in adult victims of plague from different archaeological sites, periods and latitudes.

Site	Period	Latitude	Sample (sexed adults)	Sex ratio	Freq. of Hypoplasia	Freq. of Cribra Orbitalia	Freq. of Hyperostosis	Ref.
St. Pierre de Dreux	14^th^ c.	48.737112	26	0.73	56.2	31.3	17.6	^16^
Barcelona	14^th^ c.	41.386527	13	1.17	44.4	11.1	9.1	^15^
Royal Mint	1348–49	51.50267	377	1.26	75.5	18.3	90.0	^44, 45^
Hereford	14^th^ c.	52.055908	92	0.77	89.0	13.0	33.3	^16^
Les Fedons	16^th^ c.	43.655419	56	1.15	76.4	20.0		^16^
Maria Troon	16^th^ c.	51.025078	38	1.11	75.0	36.0	24.1	^16, 85^
Puy St. Pierre	1629–30	44.53332	16	1.00	5.9			^79^

Multiple Linear Regression Models were used to test the association between sex ratio (dependent variable) of the single sites and indicators of health, period and latitude with a two-step model. In the first model, we tested which of the most studied biomarkers in our dataset, used as independent continuous variables, could be considered the best predictor of frailty associated with sex ratio: (a) Hypoplasia; (b) Cribra Orbitalia; and (c) Porotic Hyperostosis. In the second model, we selected only one of the previously tested skeletal biomarkers (Hypoplasia) as a proxy for frailty, and we considered how this predictor was associated with sex ratio when adding latitude and historical period as independent variables. In particular, we used the latitude as continuous variable, and a categorical variable, the historical period, categorized as period 1 (16^th^ c., with Les Fedons and Maria Troon), period 2 (1630, Puy St. Pierre), and period 3 (the 14^th^ c., with St. Pierre de Dreux, Barcelona, the Royal Mint and Hereford). Period 3 was used as reference period. This categorization in periods is intended to reproduce the differences in plague selectivity observed by other scholars in different outbreaks – given the limitations to distinguish between Black Death and other outbreaks of the 14^th^ c., when not explicated by historical sources. We used the “weight” option to adjust the contribution of each individual site to the outcome of the multiple regression analysis by “weighting” them in proportion to their sample size.

For each inferential analysis, the level of significance was set at 0·05. All statistical analyses were carried out with STATISTICA for Windows (version 11.0, StatSoft, Tulsa, OK).

## Results

A total of 170 publications were found by our bibliographic search. The majority (118) were rejected because the studies did not meet the inclusion criteria of this review. The remaining 52 studies are reported in Supplementary Table S1. Of these, only 25 (Table 1) met the inclusion criteria for further statistical analyses. Data on paleo-demography (sex, age) and health status (as inferred by skeletal bio-markers) were considered.

### Age and sex disparities

**Figure 2:**
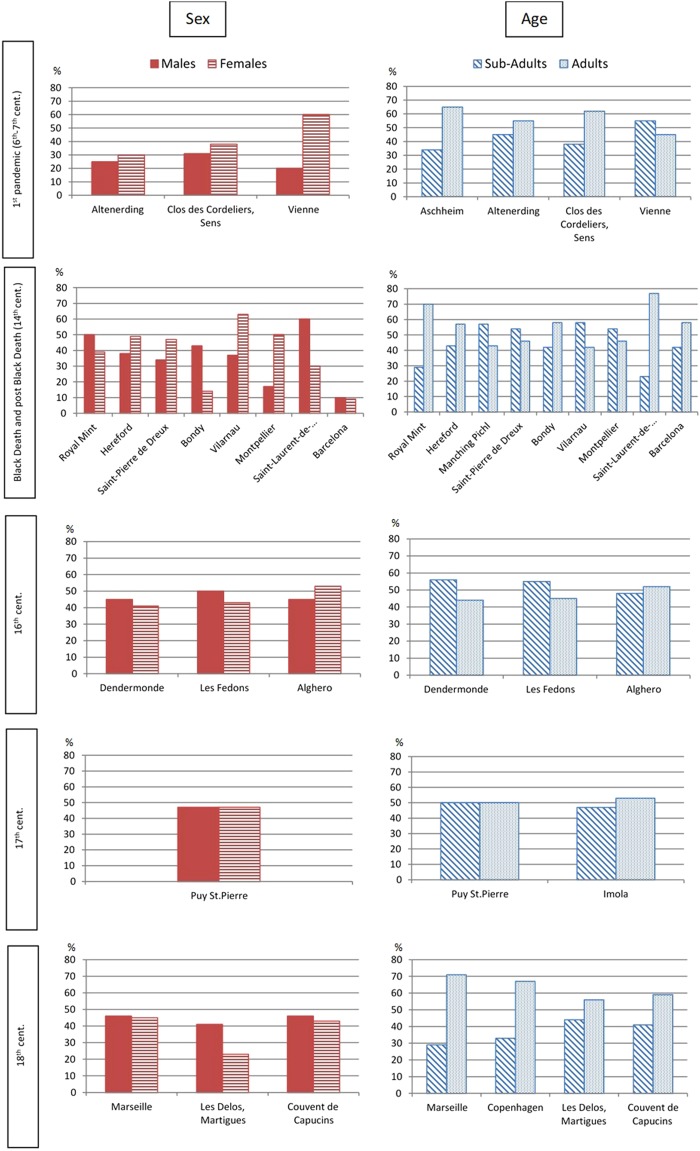
Histograms representing the frequencies of sex and age at death of plague’s victims. Samples: Aschheim(77); Altenerding (20); Clos des Cordeliers, Sens (73); Vienne (11); Royal Mint (600); Hereford (185); Manching Pichl (21); Saint-Pierre de Dreux (69); Bondy (12); Vilarnau (19); Montpellier (13); Saint-Laurent-de-la-Cabrerisse (13); Barcelona (120); Dendermonde (99); Les Fedons (133); Alghero (185); Puy St.Pierre (34); Imola (92); Marseille (179); Copenhagen (24); Les Delos, Martigues (39); Couvent de Capucins (208). Absolute values can be found in Table 1.

In Fig. 2, we reported the percent frequencies of female and male individuals in each archaeological site, subdivided by period (see also Table 1). The archaeological site of Venice could not be categorized in a period since we could not distinguish among individuals of different outbreaks (from the 14^th^ to the 17^th^ c.). From the collected data, we could not evince any particular trend due to sex-determined preferential plague lethality. No statistically significant difference was found within each group, or across the periods, between men and women. A slight prevalence of females was evident in the sites of the First Pandemic, and of males in the 16^th^ c. In all the others periods, we observed an inhomogeneous distribution of female and male individuals in the different sites. The sites of Aschheim, Manching Pichl and Imola were excluded from this test because published data on sex included not only adults but also adolescents.

The frequency distributions of sub-adults/adults appear to be more complicated (Fig. 2, Table 1), and no general trend could be observed within the different periods, with the exception of the 18^th^c., characterized by an overall prevalence of adults. As for the two classes of age at death, the differences among periods were statistically highly significant (Chi-square test, *p* = <0.001).

**Figure 3:**
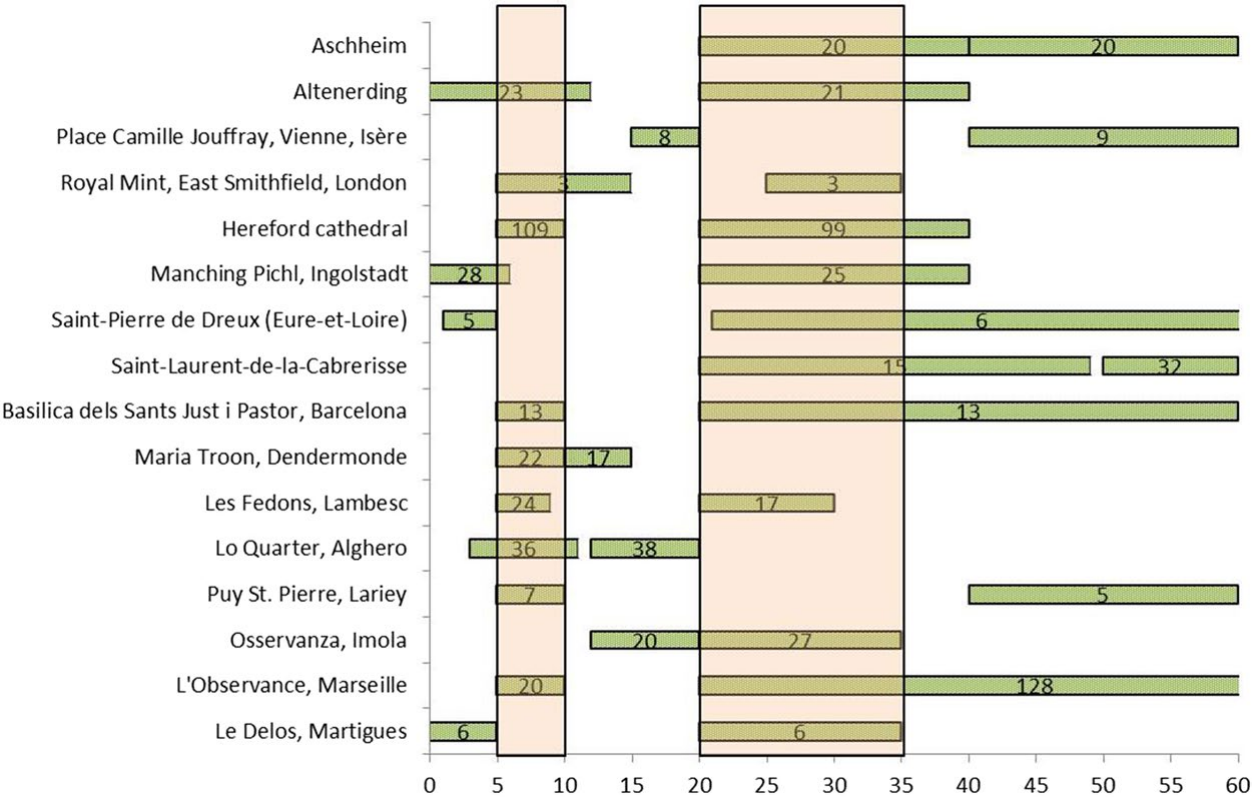
Graphical representation of the most frequent age at death classes at 16 sites with plague victims.

In Table 1, we also reported the prevalent age groups for each plague site. Moreover, in Fig. 3, we illustrate the most frequent age at death classes for each archaeological site. Apparently, the most represented classes are those of ages at death between 5–10 years and 20–35 years. Yet, any conclusion is limited by the different classes used in each study.

### Skeletal biomarkers

*Y*. *pestis* infections do not leave visible lesions on the skeleton. Thus, the traces left on the bones have a previous origin and can give insights into the health status of the victims at the time of the outbreak. Until now, very few studies have considered and described osseous lesions on plague victims.

In Supplementary Table S2, we report the most commonly observed skeletal indicators in plague victims. Linear Enamel Hypoplasia (LEH) is an unspecific stress marker that may denote malnutrition during childhood. This is the most represented marker in the examined studies. Cribra Orbitalia and Porotic Hyperostosis are usually associated with different types of anaemia: iron deficiency anaemia, megaloblastic anaemia or anaemia caused by parasitic infections^40,41^; but there are also other possible causes not connected with metabolic disorders^40^. Information on cribra was also present in several published studies, unlike that on Porotic Hyperostosis.

There are other osseous lesions occasionally considered in the studies on plague victims, which are related to bad health status of the individuals, like Harris lines in sub-adults and periosteal reactions, mainly in adults. Harris lines appear radiographically as horizontal lines or bands of increased radiopacity, mostly in the metaphysis of long bones. They are the result of slowing or total arrest of the growth of a long bone at the cartilage plate and, like LEH, are considered as evidence of exposure to elevated levels of stress in childhood^42^. Non-specific periostitis, which can be acute or chronic, occurs when the inner surface of the periosteum reacts to injury (trauma or infections) by forming woven bone that sleeves the underlying cortical bone. Short stature is also considered as a skeletal marker for biological stress during the growing phase. Though only two studies took the latter marker into account, one carried out on the sample of Royal Mint, London^43^, that reports the frequency of individuals with femur and tibia length (as a proxy measure for stature in adults) shorter than 1 standard deviation from the average observed in the sample. However, this criterion does not seem suitable for highlighting stunting conditions the cut-off of which (<3rd percentile) is far below the average of the population. The second study^17^ used the length of humerus, femur and tibia as a proxy to establish stature variation in subadults. Another study on the Royal Mint, London^44^, also considers periodontal diseases in adult individuals. Periodontitis is one of the most common chronic diseases in living populations and, according to De Witte and Bekvalac^45^, is associated with an increased risk of mortality. Finally, as suggested by other scholars, Kacki took into account also endocranial remodelling^17,46^, which is a skeletal marker of pathological status, a symptom of meningeal inflammation.

### Predictor models

We found sufficient data on skeletal biomarkers for stress only for seven plague sites (Table 2), mostly for LEH, Cribra Orbitalia and Porotic Hyperostosis. To test for the best predictor among the three indicators of health in adults, we employ Multiple Linear Regression Models. These models allow determining the relative influence of one or more predictor variables on the dependent value (in this case the sex-ratio). Moreover, they permit to identify possible outliers in the dataset. The various outcomes of the analyses are listed in Table 3.

**Table 3:** Results of Multiple Linear Regression Model.

Predictor variable	Model 1			Model 2		
	β	t	*p* value	β	t	*p* value
Cribra Orbitalia	0.163	8.178	<0.001			
Porotic Hyperostosis	0.895	48.728	<0.001			
Hypoplasia	−0.260	−13.472	<0.001	−0.741	−9.283	<0.001
Latitude				0.339	5.453	<0.001
Period						
16^th^ c. (Period 1)				1.141	8.816	<0.001
1630 (Period 2)				−1.231	−9.209	<0.001
*R* ^2^	0.831			0.135		
Adjusted *R*^2^	0.830			0.130		
*p* value	<0.001			<0.001		

All the independent variables resulted highly statistically associated with sex ratio. Among them, Cribra Orbitalia showed less influence on the fluctuations of the sex ratio across samples (β = 0.163), whereas Porotic Hyperostosis displayed the maximal linear association in this model. Apparently, this result is in contrast with the observation that Porotic Hyperostosis should not be taken as a direct indicator of health since it is only representative of a childhood condition^47–49^. In any case, we selected and analyzed only data from adults, since only in adults sex determination is reliable.

LEH showed values lower than hyperostosis, yet the association was inverse. Negative β-value (−0.260) indicates that the sex ratio enhances in populations with a low level of hypoplasia. In other words, that more males were present among plague victims with less level of stress, a quite unexpected result given the previous submissions. The overall variance of sex ratio explained by the model was 83%, meaning that the association between sex ratio and the used skeletal biomarkers is very high.

Although the level of correlation was highly significant between all the independent variables and the sex ratio, we decided to use LEH as a proxy for the health status in the second model. This choice is justified by the fact that LEH is the only variable which was reported in all the studies under examination. Another reason is that the high prevalence of Porotic Hyperostosis (especially in the Royal Mint site) can be due to a misinterpretation of the normal microporosity of the outer table of the cranial vault^50^. This normal microporosity has been reported to be more frequent in young male individuals^17,51–53^, a situation which may bias the outcomes of the analysis.

Besides, it was demonstrated that “individuals who experienced childhood stress, as evidenced by linear enamel hypoplasia, had an increased risk of death at all ages for both males and females”^47^. Thus, in plague populations with a low health status represented by high levels of LEH, we expected to find more males to have died from plague, as previously suggested. We tested here if this general consideration is true in our samples when adding another independent variable (latitude) and a categorical one, the period (Table 3). In this second model, the association between sex ratio and LEH remained negative, indicating that, in plague populations with lower frailty, males were more represented. Yet, there was just a 0.0104 decrease in sex ratio, for each unit increase in Hypoplasia. Latitude, which can be better-related to environmental gradients in Europe, was found to have a stronger effect in this model. In fact, more males died from plague in the North, with sex ratio increases of 0.0229 for each unit increase in latitude. Yet, the highest effect was linked to period change, given that by comparing the 16^th^ c. with the 14^th^ c., we detected an increase in the sex-ratio parameter, whereas a decrease was noted by comparing the 14^th^ c. and 17^th^ c. (sex ratio parameter = 0.3022 and −0.5107, respectively). This means that during the 16^th^ c. there was a higher level of males among the victims compared to the Black Death, whereas in 1630 there were more female victims, so we could not find a general trend based on the results of the multiple regression models. Yet, the fluctuation in the sex ratio over time was evidently not highly associated with the general health status of the victims, at least by using LEH as a proxy. In fact, even if all the independent variables resulted statistically associated with the sex ratio, the variance explained by the model is low (13%).

**Figure 4:**
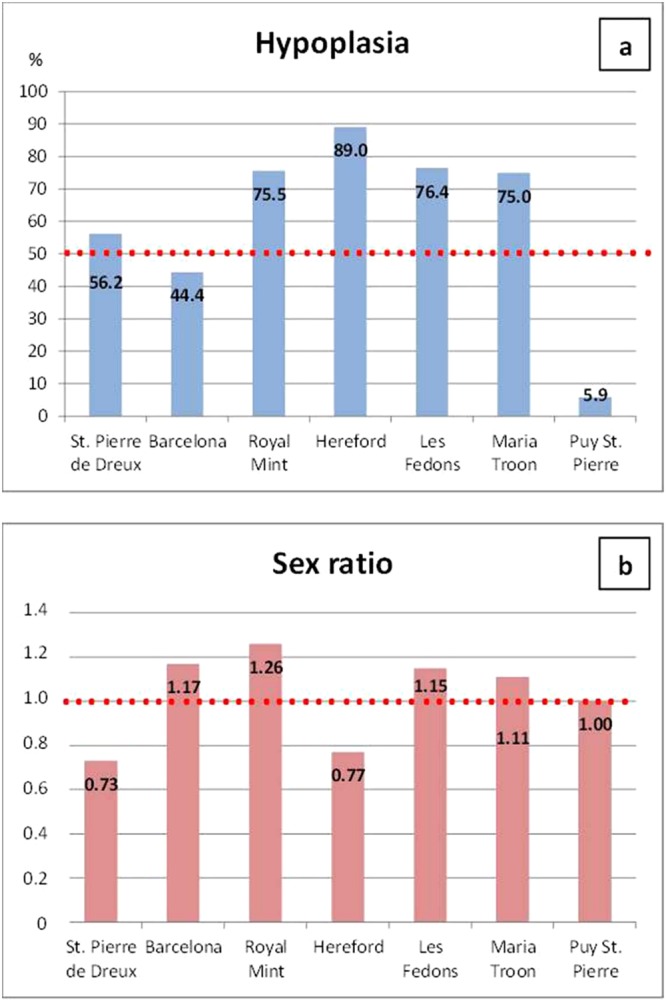
Graphical comparison of LEH-frequencies (**a**) and sex-ratio (**b**) in the same populations of plague victims. Values of sex-ratio > 1.0 indicate a prevalence of male individuals, whereas in the graph of LEH the dashed red line marks the value of 50%.

The difficulties in finding a general trend of association between LEH and sex ratio in the data are evidenced in Fig. 4, where the two variables were compared graphically for the different sites. Although the Multiple Linear Regression Models used here have been demonstrated to be accurate, no outlier was detected (see the normal probability plots of the residuals in Supplementary Fig. S1) and the correlation between the variables resulted statistically significant, they demonstrated no high linear association between the two variables, even if categorized in periods and considering their latitude. In other words, on the basis of the data published until now, it is impossible to support in general the hypothesis of plague selecting males in poor health status (LEH used as a proxy) or killing more females in a healthier population. It is also difficult to circumscribe this observation to England, as previously proposed. If for London, we could confirm with our analyses (see Fig. 4) the previously-reported trend^31^, we highlighted the opposite trend for Hereford, which most likely was also a Black Death site^54^.

## Discussion and Conclusions

By reviewing and comparing a number of anthropological studies carried out on victims of the medieval plagues, we aimed to elucidate any homogeneously prevalent risk of dying for this infectious disease in particular groups of individuals, clustered on the basis of common anthropological indicators of sex, age and health status. Certainly, some life habits and behaviours of past societies may have facilitated the spread of pestilences. In the urban environment, for instance, plague might have been favoured by overcrowding, promiscuity, poor hygienic conditions, and lack of adequate medical care^55^. Occupational exposure may also have facilitated the contact with contaminated sources, enhancing the probability of falling ill and dying from plague. Yet, it remained to be clarified whether biological and not only cultural features might have enhanced the risk of dying from this infectious disease at a global level.

Scholars have proposed that plague hit primarily people already in poor health status, and among them, males would be more likely to die^27,30^. If this were true, particular trends should be observable among plague victims of different periods and geographical regions. To adequately carry out a comparison between groups of victims retrieved in different archaeological sites, all the skeletal remains should be in similar conditions of preservation, so that markers can be recorded in a consistent fashion.

Even more importantly, the methods used for the anthropological studies should be the same, and thus comparable. This is not the case with age at death estimations and, as a result, our comparisons were strongly limited by the use of diverse age classes in the different studies. Nevertheless, the simpler distinction between subadults and adults allowed us to confirm the existence of an inhomogeneous distribution among plague victims from different populations, as previously proposed^11,34,56^, and different periods. In general, descriptive statistics showed a more abundant presence of adults, if taken globally, and we noted many peaks of distribution between 5–10 years among subadults and 20–35 years among adults (Fig. 3). A similar trend among adults was also reported by epidemiological studies carried out on plague patients at the onset of the Third Pandemic in India: “The ages most exposed to risk range between 20 and 40 years in both sexes. Thirty years seems the maximum danger point. From youth up to this figure the disease gradually increases, and having reached its height then, manifests a corresponding decline as the age advances. Plague then may be characterised as more virulent in adult life than at any other period”^57^. This observation was made in recent times, yet still in the pre-antibiotic era, thus in conditions comparable with medieval times. This evidence concerns likely cases in general and not mortality rates; only in septicemic and pneumonic plague are cases and deaths close to equal, whereas the mortality rate of not-treated bubonic plague is about 40%. Nevertheless, a higher exposure might indicate a higher mortality risk for this age range (20–40 years).

This is, seemingly, the only clear trend for plague lethality we could identify across sites and time. Similar peaks of mortality have already been highlighted in previous studies on single plague burials in France^58,59^, and in Italy^34^. Interestingly, this trend seems not to be peculiar to the Black Death, but it is present in any period and seems not to support the hypothesis of frailty due to age as an element of susceptibility to plague during the Black Death, as previously suggested^31^.

Sex-selection (against females) was proposed by some studies, which relied on documents of early modern times^32,59–63^. Anthropological data do not support this evidence as a trend for the distinct periods, with the exception of the First Pandemic, the plague burials from which were characterized by a prevalence of females. Taken together, the plague burials in our database confirm that there was no discrimination by sex in plague mortality across different populations and periods, as already proposed by the majority of epidemiological studies^20,64–67^.

In this study, we also tested the hypothesis that males were more susceptible to plague among adults grown during periods of severe malnutrition or infections, as evidenced by biological markers of stress on their skeletons. This hypothesis is also supported by demographic studies^32,68^. In our dataset of anthropological studies carried out on plague victims we did not find evidence in support of this hypothesis or of the alternative one, namely that in healthier populations, women were more frequently killed by plague. Using LEH as a proxy for health status, and Multiple Linear Regression Models we did not find any clear trend of sex-based selectivity associated with frailty across different periods and environments in Europe. However, we should underline the strong association between health status and sex ratio obtained in the first model.

Critically, there were several limitations that have affected our efforts to compare different studies. Firstly, there is a scarcity of plague sites and a lack of representative samples from different countries. Moreover, some of the anthropological analyses published until 2016 were still preliminary (eg. Manching Pichl, for which data has become available only recently^69^, and Alghero, for which the only data for sex and age determination were those produced on the field, before cleaning the skeletons). Secondly, taphonomic factors might have compromised the preservation of the most fragile remains, especially skeletons of small children and female individuals, with unpredictable effects on the ratio between sexes and between age classes. The choice, in some cases^13,70^, of the best-preserved individuals for anthropological analysis, might constitute an additional major bias. Thirdly, it should be noted that inconsistency among the methods used to determine age and sex in different studies could somehow influence comparisons. Fourthly, a retrospective diagnosis of plague should be based on the study of multiple genetic loci or, better, of high-throughput sequencing of *Y*. *pestis* DNA, even if the retrieved genome is only partial since this kind of analysis is the only one, which permits the identification of the retrieved molecules beyond a reasonable doubt. A discussion is ongoing on the reliability of some molecular tests previously used for plague diagnosis^71^. Lastly, previous pathologies of the individuals, as well as dissimilarities in the anthropological methods used to assess them, can affect the reliability of the data.

Nevertheless, this effort to overview a large number of bioarchaeological data has shown that possible generalizations about trends of plague lethality observed at single sites are not reliable and can instead be related to population composition or cultural aspects, like enhanced exposure due to work activities, family care, differences in eating habits and so on^72,73^. Genetic features, which might have affected susceptibility and resistance to plague in populations of the past are not completely explored and, for instance, one study has inferred signals of convergent evolution likely shaped by plague in modern populations^74^. Yet, more ancient DNA studies on victims of past epidemics would be required to directly address the question by exploring signatures of selection across time, and not only for plague but also for other infectious diseases, as previously proposed^75,76^.

The extension of the global approach here proposed to other relevant epidemic diseases of the past will enable, in the near future, wider comparisons and the identification of possible biological trends and patterns, which might be specifically linked to a single infectious disease. This will consistently reinforce our understanding of host-pathogen interactions, particularly in the case of those triggered at an organismal or population level.

## Electronic supplementary material

Electronic Supplementary Material
