# Supplementary material for: A critical review of anthropological studies on skeletons from European plague pits of different epochs
Source: Sci Rep. 2018 Dec 5;8:17655. doi: 10.1038/s41598-018-36201-w (PMC6281611; doi:10.1038/s41598-018-36201-w)
Supplement: Supplementary file 1 — Electronic Supplementary Material [file 41598_2018_36201_MOESM1_ESM.pdf]

Electronic Supplementary Material

A critical review of anthropological studies on skeletons from European plague pits of different epochs.

Bramanti B[1,2,3] , Zedda N[1]*, Rinaldo N[1]*, Gualdi-Russo E[1]

[1]Department of Biomedical and Specialty Surgical Sciences, University of Ferrara, Ferrara, Italy

[2]Centre for Ecological and Evolutionary Synthesis, Department of Biosciences, University of Oslo, Oslo, Norway

[3]University Center for Studies on Gender Medicine, University of Ferrara, Ferrara, Italy

*Corresponding authors

Supplementary Table S1

Studies on skeletal remains of plague victims

Author	Site	Country	Period		n° ind.	Burial Type*	Basis of Plague determination			Anthropological studies				note	Included
			Date	C^{14}			DNA	Immun.	Historical texts	sex	age	pathologies	Biological Stress markers		
Staskiewiz 2007 [1]	Aschheim	Germany	5th-7th cent.	530-630 [2]	77	Mult	✓ [3]			✓	✓	✓			x
Seifert 2014 [2]PhD thesis	Aschheim	Germany	5th-7th cent.	530-630	19	Mult	✓ [3]			✓	✓				
Helmuth and Ankner, 1996[4]	Altenerding	Germany	5th-7th cent.		20	Ind+Mult	✓ [3]			✓	✓			1	x
Castex, 2007[5]	Le Clos des Cordeliers, Sens, Yonne	France	5th-6th cent.		68	Mult	✓ [6]	✓ [7]			✓			Graph with mortality rates	
Castex, 2008[8]	Le Clos des Cordeliers, Sens, Yonne	France	5th-6th cent.		73	Mult	✓ [6]	✓ [7]		✓	✓			Graph with mortality rates	
Castex, Kacki, 2016 [9]	Le Clos des Cordeliers, Sens, Yonne	France	5th-6th cent.		73	Mult	✓ [6]	✓ [7]		✓	✓				x
Castex, Kacki, 2016 [9]	Poitiers, Poitou-Charentes	France	5th-6th cent.		53	Mult									
Signoli et al. 2009 [10]	Place Camille Jouffray, Vienne (Isère)	France	760-880	610-1040	11	Mult	✓ [11]	✓ [12]		✓	✓				x
Rubini 1991[13]	Castro dei Volsci, Frosinone	Italy	6th cent.		148	Mult				✓	✓	✓	✓		
Rubini et al. 2016[14]	Castro dei Volsci, Frosinone	Italy	6th cent.		179	Mult				✓	✓				
Waldron 1992[15]	Royal Mint, East Smithfield, London	UK	1348		600	Mult	✓ [16]			✓	✓	✓			x
Waldron 2001[17]	Royal Mint, East Smithfield, London	UK	1348		600	Mult	✓ [16]			✓	✓	✓			x

Author	Site	Country	Period		n° ind.	Burial Type*	Basis of Plague determination			Anthropological studies				note	Included
			Date	C^{14}			DNA	Immun.	Historical texts	sex	age	pathologies	Biological Stress marker		
Margerison and Knüsel, 2002[18]	Royal Mint, East Smithfield, London	UK	1349		473	Mult	✓ [16]			✓	✓				
Gowland and Chamberlain, 2005[19]	Royal Mint, East Smithfield, London	UK	1348		600	Mult	✓ [16]				✓			Graph with percentage of age classes	
DeWitte, 2006[20] PhD thesis	Royal Mint, East Smithfield, London	UK	14th cent.		491	Mult	✓ [16]			✓	✓	✓	✓		
DeWitte, 2010[21]	Royal Mint, East Smithfield, London	UK	14th cent.		299	Mult	✓ [16]			✓		✓	✓		
DeWitte, 2010b[22]	Royal Mint, East Smithfield, London	UK	14th cent.		337	Mult	✓ [16]				✓			Graph with percentage of age classes	
De Witte 2012[23]	Royal Mint, East Smithfield, London	UK	14th cent.		161	Mult	✓ [16]				✓	✓		analysis of periodontal diseases	x
DeWitte and Hughes-Morey, 2012[24]	Royal Mint, East Smithfield, London	UK	14th cent.		127	Mult	✓ [16]			✓			✓		
De Witte, Slavin 2013[25]	Royal Mint, East Smithfield, London	UK	14th cent.		491	Mult	✓ [16]						✓		x
Rubini et al. 2016[14]	Royal Mint, East Smithfield, London	UK	1348-1350		636	Mult	✓ [16]				✓			Graph with percentage of age classes	
Grainger and Phillpotts, 2011[26]	St Mary Grace Abbey, East Smithfield, London	UK	1350-1400		199	Ind	✓ [3,16]			✓	✓	✓	✓		
Kacki 2016[27] PhD thesis	Hereford cathedral	UK	14th cent.	1281-1389[28]	185	Mult	✓ [28]	✓ [28]		✓	✓	✓	✓		x
Castex, Kacki, 2016[29]	Hereford cathedral	UK	14th cent.	1281-1389[28]	185	Mult	✓ [28]	✓ [28]		✓	✓				
Prechel 1996[30]	Lubeck	Germany	14th cent.	1260-1390	671	Mult				✓	✓				

Author	Site	Country	Period		n° ind.	Burial Type*	Basis of Plague determination			Anthropological studies				note	Included
			Date	C^{14}			DNA	Immun.	Historical texts	sex	age	pathologies	Biological Stress marker		
Lütgert, 2000[31]	Lubeck	Germany	14th cent.	1260-1390	816	Mult				✓	✓			not all are plague's victims	
Wiechmann et al. 2010[32]	Manching Pichl, Ingolstadt	Germany	1250-1500		6	Mult	✓[32]			✓	✓				
Seifert 2014[2] PhD thesis	Manching Pichl, Ingolstadt	Germany	1250-1500		21	Mult	✓[32]			✓	✓				x
Castex 2007[5]	Saint-Pierre de Dreux (Eure-et-Loire)	France	14th cent.		72	Mult	✓[6]				✓			Graph with mortality rates	
Castex 2008[8]	Saint-Pierre de Dreux (Eure-et-Loire)	France	14th cent.		72	Mult	✓[6]			✓	✓			Graph with mortality rates	
Castex, Kacki, 2016[29]	Saint-Pierre de Dreux (Eure-et-Loire)	France	14th cent.		72	Mult	✓[6]			✓	✓				
Kacki 2016[27] PhD thesis	Saint-Pierre de Dreux (Eure-et-Loire)	France	14th cent.		69	Mult	✓[6]			✓	✓	✓	✓		x
Passarius et al. 2008[33]	Vilarnau	France	14th cent.		19	Mult	✓			✓	✓				x
Le Forestier 2012[34]	Bondy	France	14th cent.	1297-1373	12	Mult	✓[35]	✓[7]		✓					x
Kacki et al., 2011[36]	Saint-Laurent-de-la-Cabrerisse	France	14th cent.	1279-1409	13	Ind+Mult	✓[28]	✓		✓	✓				x
Crubezy et al. 2006[37]	Saints-Côme-et-Damien, Montpellier	France	14th cent.		13	Mult	✓[38]			✓	✓				x
Kacki, Castex 2004[39]	Basilica dels Sants Just i Pastor, Barcelona	Spain	14th cent.	14th cent.[40]	120	Mult	✓[41]			✓	✓	✓	✓		x
Castex,Kacki, 2016[29]	Basilica dels Sants Just i Pastor, Barcelona	Spain	14th cent.	14th cent.[40]	120	Mult	✓[41]			✓	✓				

Author	Site	Country	Period		n° ind.	Burial Type*	Basis of Plague determination			Anthropological studies				note	Included
			Date	C^{14}			DNA	Immun.	Historical texts	sex	age	pathologies	Biological Stress marker		
Gambaro et al. 2001[42]	Lazzaretto Vecchio, Venice	Italy	14th-17th cent.		331	Mult	✓ [43]	✓ [7]	✓		✓				x
Signoli et al., 2008[44]	Lazzaretto Vecchio,Venice	Italy	1478-1486		184	Mult	✓ [43]	✓ [7]	✓		✓				
Bizot 2005[45]	Les Fedons, Lambesc	France	1590		133	Ind+Mult	✓ [46]	✓ [12]	✓	✓	✓				
Castex 2007[5]	Les Fedons, Lambesc	France	16th cent.		133	Ind+Mult	✓ [46]	✓ [12]	✓ [45]		✓			Graph with mortality rates	
Castex 2008[8]	Les Fedons, Lambesc	France	16th cent.		133	Ind+Mult	✓ [46]	✓ [12]	✓ [45]	✓	✓			Graph with mortality rates	
Bianucci et al. 2008[12]	Les Fedons, Lambesc	France	1590		133	Ind+Mult	✓ [46]	✓	✓ [45]	✓					
Kacki 2016[27] PhD thesis	Les Fedons, Lambesc	France	16th cent.		133	Ind+Mult	✓ [46]	✓ [12]	✓ [45]	✓	✓	✓	✓		x
Castex, Kacki, 2016[29]	Les Fedons, Lambesc	France	16th cent.		133	Ind+Mult	✓ [46]	✓ [12]	✓ [45]	✓	✓				
Bianucci et al., 2009[47]	Sainte-Croix Abbey, Poitiers	France	After 1524		6	Ind		✓		✓	✓				
Boucherie et al. 2016[48]	Maria Troon, Dendermonde	Belgium	1579-1584	1455-1632[27]	99	Ind+Mult			✓ [49]	✓	✓		✓		x
Kacki 2016[27] PhD thesis	Maria Troon, Dendermonde	Belgium	16th cent.	1455-1632[27]	99	Ind+Mult			✓ [49]	✓	✓	✓	✓		x
Castex, Kacki, 2016[29]	Maria Troon, Dendermonde	Belgium	16th cent.	1455-1632[27]	99	Ind+Mult			✓ [49]	✓	✓				
Milanese, 2010[50]	Lo Quarter, Alghero	Italy	1582-1583		185	Mult		✓ [51]		✓	✓				x
Bianucci et al. 2012[51]	Lo Quarter, Alghero	Italy	1582-1583		10	Mult		✓		✓	✓				
Seifert 2014[2] PhD thesis	Brandenburg	Germany	1618–1648		9	Mult	✓			✓	✓				
Signoli et al. 2007[52]	Puy St. Pierre, Lariey	France	17th cent.		34	Ind+Mult		✓ [12]		✓	✓				
Bianucci et al. 2008[12]	Puy St. Pierre, Lariey	France	1628-1632		34	Ind+Mult		✓		✓	✓				
Ardagna et al. 2012[53]	Puy St. Pierre, Lariey	France	1629-1630		34	Ind+Mult		✓ [12]		✓	✓	✓	✓		x
Bianucci et al. 2009	La Chaize-le-Vicomte	France	1600-1700		6	Mult		✓		✓	✓				

Author	Site	Country	Period		n° ind.	Burial Type*	Basis of Plague determination			Anthropological studies				note	Included
			**Date**	**C^{14}**			**DNA**	**Immun.**	**Historical texts**	**sex**	**age**	**pathologies**	**Biological Stress marker**		
Bianucci et al. 2008	La Butte aux Herbes, Draguignan	France	1649-1650		8	Mult	✓	✓		✓	✓				
Hadjouis et al., 2006[54]	Saint-Maurice, Charenton le Pont	France	17th cent.		3	Ind	✓				✓	✓	✓		
Rinaldo et al. 2014[55]	Osservanza, Imola	Italy	1629-1630		92	Mult		✓ [14]	✓ [56]	✓	✓	✓	✓	Only dental pathologies	x
Rubini et al. 2016[14]	Osservanza, Imola	Italy	1629-1630		114	Mult		✓ [14]	✓ [56]		✓				
Caruso et al., 2013[57]	Viale Sabotino, Milan	Italy	17th cent.		240	Mult				✓	✓	✓	✓		
Fiscella et al. 2008[67]	Copenhagen	Denmark	1711-1712		24	Ind+Mult			✓		✓		✓		x
Dutour et al. 1994[58]	Rue Leca, Marseille	France	1720-1722		22	Mult	✓ [59]	✓ [12]	✓	✓	✓				
Signoli and Dutour, 1997[60]	Rue Leca, l'Observance, Marseille	France	1720-1722		22	Mult	✓ [59]	✓ [12]	✓ [58]	✓	✓				x
Signoli and Dutour, 1998[60]	Rue Leca, l'Observance, Marseille	France	1720-1722		303	Mult	✓ [59]	✓ [12]	✓ [58]		✓				
Bello et al. 2006[61]	L'Observance, Marseille	France	1722		179	Mult	✓ [59]	✓ [12]	✓ [58]	✓	✓				x
Bianucci et al. 2008[12]	L'Observance, Marseille	France	1722		172	Mult	✓ [59]	✓	✓ [58]						
Signoli et al., 2002[62]	L'Observance, Marseille	France	1720		216	Mult	✓ [59]	✓ [12]	✓ [58]		✓			Graph with percentage of age classes	
Signoli, 2008[63]	L'Observance, Marseille	France	1720		216	Mult	✓ [59]	✓ [12]	✓ [58]		✓			Graph with percentage of age classes	
Chaumoitre et al. 2007[64]	Delos, Martigues + L'observance, Marseille	France	1720-1722		113	Mult	✓ [59]	✓ [12]	✓ [58,65]		✓		✓		x
Signoli et al., 1995[65]	Le Delos, Martigues	France	1720-1722		39	Mult		✓ [12]	✓ [65]	✓	✓				

Bianucci et al. 2008[12]	Le Delos, Martigues	France	1720-1722		39	Mult		✓[12]	✓[65]	✓	✓				x
Bianucci et al. 2008[12]	Le Couvent des Capucins de Ferrieres, Martigues	France	1720-1722		210	Mult	✓[11]	✓	✓[66]						
Tzortzis, Signoli 2009[66]	Le Couvent des Capucins de Ferrieres, Martigues	France	1720-1722		208	Mult	✓[11]	✓[12]	✓	✓	✓				x

[1]In Feldman et al. (2016), to seek for *Y. pestis*' DNA, the authors use samples from 10 out of the 16 double burials found in the early-medieval (5th-7th century) cemetery of Altenerding. From the names of the burials reported in the supplementary information of the paper, we could reconstruct the sex attribution and age-at-death of all the individual in a catalogue[4]. Yet, in the same catalogue, the 20 inhumations are attributed to different periods (6th and 7th c.). Since only two individuals (1175, 1176) were positive to molecular analyses, we cannot be sure that all the 20 individuals died for plague. For this reason, we have decided to exclude the site from the statistics, although a genome of the etiological agent is published.

Supplementary Table S2

Studies on skeletal remains of plague victims: skeletal bio-markers

Skeletal Markers of Biological Stress

Geographic area	Site	Period	Sample n.	Low Stature	Cribra Orbit.	Porotic Hyperost.	Hypopl.	Harris lines	Periostitis	Endocr. Remodeling	Osteoarthritis	Co-morbidities %	Ref.
Southern Germany	Aschheim	5th-7th cent.	77		12/77		7/77						[1]
UK	Royal Mint, East Smithfield, London	14th	491	A 5/ 44	A 17/ 93	A 85/ 94	A 74/ 98					Periodontal disease: 80/ 81	[23,25]
UK	Royal Mint, East Smithfield, London	1348-1349	600								15-45+: M=(29.9%) F= (19.4%)		[15]
UK	Hereford	14th	185		A 4/31 S 16/38	A 12/36 S 7/50	A 33/37 S 39/48		A 32/78 S 6/40	2/88			[27]
Northern France	Saint-Pierre de Dreux (Eure-et-Loire)	14th	69		A 5/16 S 10/16	A 5/ 23 S 3/17	A 9/16 S 9/15		A 8/16 S 4/19	A 1/13 S 5/ 30			[27]
Spain	Basilica del Sants Just i Pastor, Barcelona	14th	120		A 1/ 9 S 2/10	A 1/11 S 1/13	A 4/ 9 S 9/15					Caries: 2/ 9	[39]
Belgium	Maria Troon, Dendermonde	16th	99		A 9/25 S 12/23	A 7/29 S 8/30	A 15/20 S 24/30	36/ 69	A 8/24 S 16/27	A 4/24 S 2/21			[27,48]

Southern France	Les Fedons, Lambesc	16th	133		A 9/45<br>S 13/30		A 42/55<br>S 34/53			S 1/ 34			27
Southern France	Puy St. Pierre, Lariey	1629-1630	34				A 1/17<br>S 2/17		A 2/17<br>S 4/17			Tubercolosis:<br>1/ 34	53
Northern Italy	Osservanza, Imola	1629-1630	92				51/ 92					Caries: 65%<br>Tartar: 53%<br>Abscess:9%	55
Denmark	Copenhagen	1711-1712	24					3/ 24					67
Southern France	Delos, Martigues + L'observance, Marseille	1720-1722	113					72/ 113					64

A=Adult; S=SubAdult

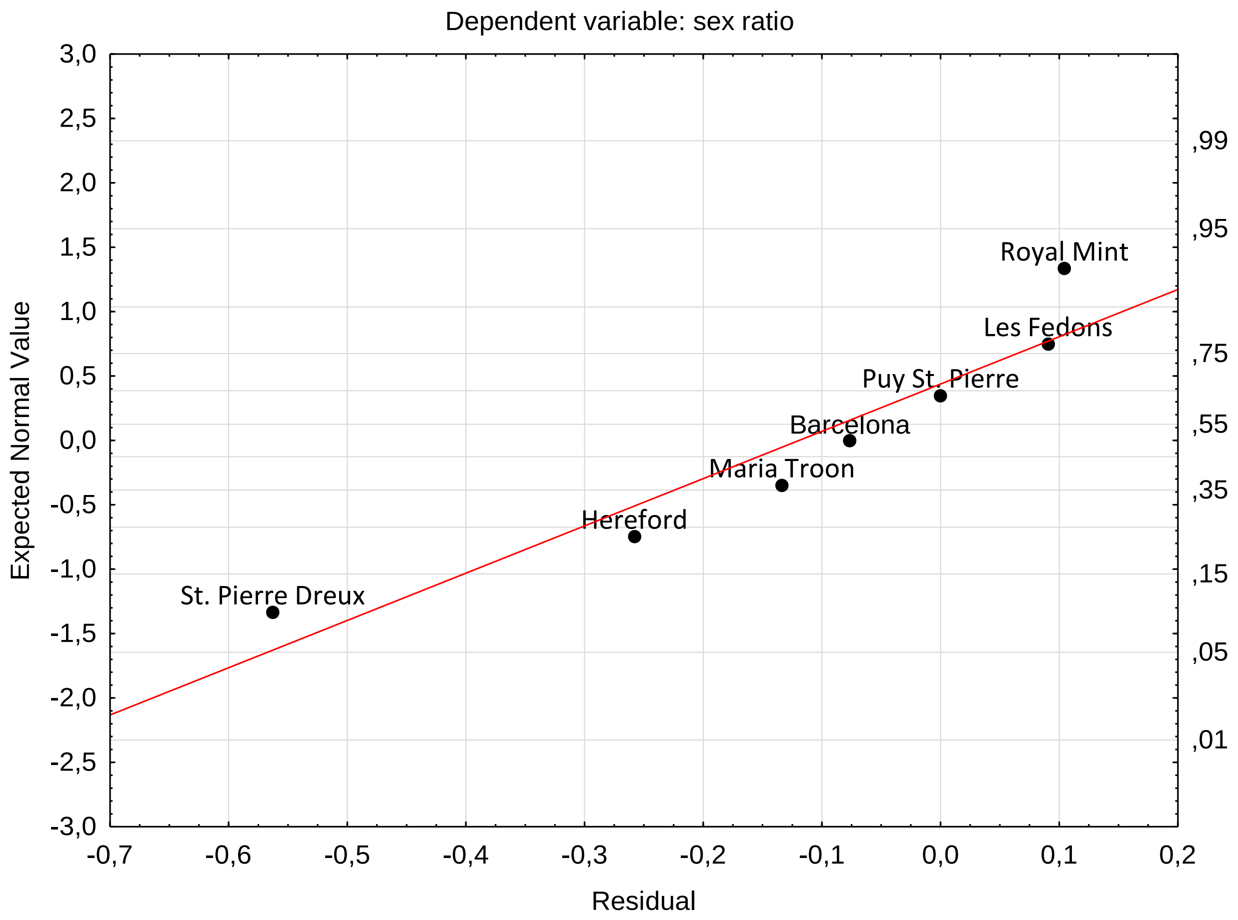

Supplementary Figure S1. Normal Probability Plot of Raw Residuals

References

1. Staskiewicz, The early medieval cemetery at Aschheim-Bajuware. in *Skeletal series and their socioeconomic context. Documenta Archaeobiologiae 5* (eds Grupe, G. & Peters, J.) 35–56 (Verl. M. Leidorf, Rahden, 2007).

2. Seifert, L. *Mikroevolution und Geschichte der Pest: paläogenetische Detektion und Charakterisierung von Yersinia pestis, gewonnen aus historischem Skelettmaterial.* PhD thesis, Ludwig-Maximilians-Universität München (2014).

3. Feldman, M. *et al.* A High-Coverage Yersinia pestis Genome from a Sixth-Century Justinianic Plague Victim. *Mol. Biol. Evol.* **33,** 2911–2923 (2016).

4. Helmuth, H. & Ankner, D. *Das Reihengräberfeld von Altenerding in Oberbayern: Anthropologie, Damaszierung und Textilfunde* (von Zabern, Mainz, 1996).

5. Castex, D. De la composition par âge et par sexe des populations archéologiques à l'identification des grandes épidémies du passé. *CUDEP Bordeaux, Séance 4 - Dynamique des épidémies* **4,** (2007).

6. Drancourt, M. *et al.* Genotyping, Orientalis-like Yersinia pestis, and plague pandemics. *Emerg. Infect. Dis.* **10,** 1585 (2004).

7. Malou, N. *et al.* Immuno-PCR - A new tool for paleomicrobiology: The plague paradigm. *PLoS One* **7,** 10.1371/journal.pone.0031744 (2012).

8. Castex, D. Identification and interpretation of historical cemeteries linked to epidemics. *Paleomicrobiology past Human Infections* (eds Raoult, D. & Drancourt, M.), 23–48 (Springer, Berlin, 2008).

9. Castex, D. & Kacki, S. Demographic Patterns Distinctive of Epidemic Cemeteries in Archaeological Samples. *Microbiol. Spectr.* **4,** (2016).

10. Rigeade, C. & Bizot, B., Le Bot-Helly, A, Signoli, M. Une sépulture de pestiférés du Haut Moyen Âge à Vienne (Isère). *Archéologie du Midi médiéval* **27,** 19–29 (2009).

11. Drancourt, M. *et al.* Yersinia pestis Orientalis in remains of ancient plague patients. *Emerg. Infect. Dis.* **13,** 332–333 (2007).

12. Bianucci, R. *et al.* Technical note: A rapid diagnostic test detects plague in ancient human remains: An example of the interaction between archeological and biological approaches (southeastern France, 16th-18th centuries). *Am. J. Phys. Anthropol.* **136,** 361–367 (2008).

13. Rubini, M. *La necropoli di castro dei Volsci: problematiche ed aspetti di antropologia fisica*. (Ministero per i Beni Culturali e Ambientali, Roma, 1991).

14. Rubini, M., Gualdi-Russo, E., Manzon, V. S., Rinaldo, N. & Bianucci, R. Mortality risk factors show similar trends in modern and historic populations exposed to plague. *J. Infect. Dev. Ctries.* **10,** 488–493 (2016).

15. Waldron, T. Osteoarthritis in a Black Death cemetery in London. *Int. J. Osteoarchaeol.* **2,** 235–240 (1992).

16. Bos, K. I. *et al.* A draft genome of Yersinia pestis from victims of the Black Death. *Nature* **478,** 506–510 (2011).

17. Waldron, H. A. Are plague pits of particular use to palaeoepidemiologists? *Int. J. Epidemiol.* **30,** 104–108 (2001).

18. Margerison, B. J. & Knüsel, C. J. Paleodemographic comparison of a catastrophic and an attritional

death assemblage. *Am. J. Phys. Anthropol.* **119,** 134–143 (2002).

19. Gowland, R. & Chamberlain, A. T. Detecting plague : palaeodemographic characterisation of a catastrophic death assemblage. *Antiquity* **79,** 146–157 (2005).

20. DeWitte, S. N. *The paleodemography of the Black Death 1347-1351*. PhD Thesis, Pennsylvania State University (2006).

21. DeWitte, S. N. Sex differentials in frailty in medieval England. *Am. J. Phys. Anthropol.* **143,** 285–297 (2010).

22. DeWitte, S. N. Age patterns of mortality during the Black Death in London, AD 1349–1350. *J. Archaeol. Sci.* **37,** 3394–3400 (2010).

23. Dewitte, S. N. Sex differences in periodontal disease in catastrophic and attritional assemblages from medieval london. *Am. J. Phys. Anthropol.* **149,** 405–416 (2012).

24. DeWitte, S. N. & Hughes-Morey, G. Stature and frailty during the Black Death: the effect of stature on risks of epidemic mortality in London, AD 1348–1350. *J. Archaeol. Sci.* **39,** 1412–1419 (2012).

25. Dewitte, S. & Slavin, P. Between Famine and Death : England on the Eve of the Black Death — Evidence from Paleoepidemiology and Manorial Accounts. *J. Interdiscip. Hist.* **44,** 37–60 (2013).

26. Grainger, I. & Phillpotts, C. *The Cistercian abbey of St Mary Graces, East Smithfield, London*. (Museum of London Archaeology, London, 2011).

27. Kacki, S. *Influence de l'état sanitaire des populations anciennes sur la mortalité en temps de peste. Contribution à la paléoépidémiologie*. PhD Thesis, University of Bordeaux, 2016).

28. Haensch, S. *et al.* Distinct clones of Yersinia pestis caused the Black Death. *PLoS Pathog.* **6,** 10.1371/journal.ppat.1001134 (2010).

29. Castex, D. & Kacki, S. Demographic Patterns Distinctive of Epidemic Cemeteries in Archaeological Samples. *Microbiol. Spectr.* **4,** (2016).

30. Prechel, M. Anthropologische Untersuchungen der Skelettreste aus einem Pestmassengrab am Heiligen-Geist-Hospital zu Lübeck. *Lübecker Schriften zur Archäologie und Kult.* 3232–3339 (1996).

31. Lütgert, S. A. Victims of the Great Famine and the Black Death? The archaeology of the mass graves found in the former graveyard of Holy Ghost Hospital, Lubeck (N. Germany), in the European context. *Hikuin* **27,** 255–264 (2000).

32. Wiechmann, I., Harbeck, M. & Grupe, G. Yersinia pestis DNA sequences in late medieval skeletal finds, Bavaria. *Emerg. Infect. Dis.* **16,** 1806 (2010).

33. Passarrius, O., Donat, R. & Catafau, A. *Vilarnau: un village du Moyen Âge en Roussillon*. (Éditions Trabucaire, Perpignan, 2008).

34. Le Forestier, C. La Peste Noire à Bondy. *Bulletin de la Société historique du Raincy et du Pays de l'Aulnoye* **41,** 5-13 (2012).

35. Tran, T. N. N., Le Forestier, C., Drancourt, M., Raoult, D. & Aboudharam, G. Brief communication: Co-detection of Bartonella quintana and Yersinia pestis in an 11th-15th burial site in Bondy, France. *Am. J. Phys. Anthropol.* **145,** 489–494 (2011).

36. Kacki, S., Rahalison, L., Rajerison, M., Ferroglio, E. & Bianucci, R. Black Death in the rural cemetery of Saint-Laurent-de-la-Cabrerisse Aude-Languedoc, southern France, 14th century: Immunological evidence. *J. Archaeol. Sci.* **38,** 581–587 (2011).

37. Crubézy, E., Duchesne, S. & Arlaud, C. *La mort, les morts et la ville (Montpellier–Xe-XVIe siècles).*

(Editions Errance, Paris, 2006).

38. Raoult, D. *et al.* Molecular identification by 'suicide PCR' of Yersinia pestis as the agent of medieval black death. *Proc. Natl. Acad. Sci. U. S. A.* **97,** 12800–3 (2000).

39. Kacki, S. & Castex, D. La sépulture multiple de la basilique des Saints Martyrs Just et Pastor : bio-archéologie des restes humains. *Quad. d'Arqueologia i Història la Ciutat Barcelona* **10,** 180–199 (2014).

40. Beltrán de Heredia, J. & Gibrat, I. El primer testimoni arqueològic de la Pesta Negra a Barcelona: La fossa comuna de la basílica dels Sants Màrtirs Just i Pastor. *Quad. D'Arqueologia I Història La Ciutat Barcelona* **10**, 164–179 (2014).

41. Spyrou, M. A. *et al.* Historical Y. pestis Genomes Reveal the European Black Death as the Source of Ancient and Modern Plague Pandemics. *Cell Host Microbe* **19,** 874–881 (2016).

42. Gambaro, L. *et al.* La fouille de l'île du Lazzaretto Vecchio de Venise: premières données. in *La peste: entre épidémies et sociétés* (eds Signoli, M., Chevé, D., Adalian, P., Boëtsch, G. & Dutour, O.) 97–103 (Firenze University Press, Firenze, 2001) 97–103 (Firenze University Press, Firenze, 2001).

43. Tran, T. N. N. *et al.* High throughput, multiplexed pathogen detection authenticates plague waves in medieval Venice, Italy. *PLoS One* **6,** 1–5, 10.1371/journal.pone.0016735 (2011).

44. Signoli, M., Gambaro, L., Rigeade, C. & Drusini, A. Les fouilles du Lazzareto Vecchio (Venise, Italie). in *9e journées d'anthropologie de Valbonne* (eds Buchet, L., Rigeade, C., Séguy, I., Signoli, M.) 333–346 (Editions ADPCA, Antibes, 2008).

45. Bizot, B., Castex, D., Reynaud, P., Signoli, M. *La Saison d'une peste (avril-septembre 1590): Le cimetière des Fédons à Lambesc (Bouches-du-Rhône).* (CNRS Éditions, Paris, 2005).

46. Drancourt, M., Aboudharam, G., Signoli, M., Dutour, O. & Raoult, D. Detection of 400-year-old Yersinia pestis DNA in human dental pulp: An approach to the diagnosis of ancient septicemia. *Proc. Natl. Acad. Sci. USA* **95,** 12637–12640 (1998).

47. Bianucci, R. *et al.* Plague immunodetection in remains of religious exhumed from burial sites in central France. *J. Archaeol. Sci.* **36,** 616–621 (2009).

48. Boucherie, A., Castex, D., Polet, C. & Kacki, S. Normal growth, altered growth? Study of the relationship between harris lines and bone form within a post-medieval plague cemetery (Dendermonde, Belgium, 16th Century). *Am. J. Hum. Biol.* **29,** (2016).

49. Goudie Falkenbach E; Ryssaert C, Brion M, Castex D, Rouzic M, Colombo A, K. D. Archeologisch onderzoek in Dendermonde op de site van het voormalige Birgitinessenklooster Maria Troon. *Archelog Mediev.* **35,** 142–149 (2012).

50. Milanese, M. *Lo scavo del cimitero di San Michele ad Alghero (fine XIII–inizi XVII secolo).* (Felici Ed., Pisa, 2010).

51. Bianucci, R., Giuffra, V., Ferroglio, E., Milanese, M. & Fornaciari, G. ' Lo Quarter': the Alghero plague cementery (1582-1583 AD). *Journal of Biological Research della Società Italiana di Biologia Sperimentale* **85,** (2012).

52. Signoli, M. *et al.* Découverte d'un cimetière de pestiférés du xviie siècle (Puy-Saint-Pierre, Hautes-Alpes, France). in *Peste: entre épidémies et sociétés.* (ed. M. Signoli, D. Chevé, P. Adalian, G. Boëtsch, O. Dutour) 131–135 (Firenze University Press, Firenze, 2007).

53. Ardagna, Y., Tzortzis, S., Bizot, B. & Signoli, M. Profil paléopathologique d'un cimetière de pestiférés du XVIIe siècle (Puy-Saint-Pierre, Hautes-Alpes, France). *Antropo* **27,** 63–72 (2012).

54. Hadjouis, D., LA VU, D., Aboudharam, G., Drancourt, M. & Andrieux, P. Présence de la peste (Yersinia pestis) dans le cimetière protestant de Saint-Maurice au XVIIème siècle (Val-de-Marne, France). Archéologie et microbiologie. *Paleobios*, 14 (2006).

55. Rinaldo, N., Manzon, V. S., Muro, X. G. & Gualdi-russo, E. La peste del 1630: analisi antropologiche preliminari dei resti scheletrici provenienti dal complesso dell' O sservanza di Imola. *Ann. dell'Università di Ferrara Museol. Sci. e Nat.* **10,** 135–140 (2014).

56. Cervellati, I. La comunità imolese e la peste del 1630-2. in *Pagine di vita e di storie imolesi* (Ed. Cars, Imola, 1986).

57. Caruso, V. *et al.* Gli scheletri della fossa comune di viale Sabotino a Milano: le vittime della peste manzoniana? *The Journal of Fasti Online* **2,** 1–11 (2013).

58. Dutour, O., Signoli, M., Georgeon, E. & DA SILVA, J. Le charnier de la Grande Peste de Marseille (rue Leca): données de la fouille de la partie centrale et premiers résultats anthropologiques. *Préhistoire Anthropol. méditerranéennes* **3,** 191–203 (1994).

59. Bos, K. I. *et al.* Eighteenth century Yersinia pestis genomes reveal the long-term persistence of an historical plague focus. *Elife* **5,** 1–11 10.7554/eLife.12994 (2016).

60. Signoli, M. & Dutour, O. Le charnier du couvent de l'Observance: 1722. *Provence Hist. fasc* **189,** 469–488 (1998).

61. Bello, S. M., Thomann, A., Signoli, M., Dutour, O. & Andrews, P. Age and sex bias in the reconstruction of past population structures. *Am. J. Phys. Anthropol.* **129,** 24–38 (2006).

62. Signoli, M., Séguy, I., Biraben, J.-N., Dutour, O. & Belle, P. Paleodemography and historical demography in the context of an epidemic. *Population (Paris).* **57,** 829–854 (2002).

63. Signoli, M. Archéo-anthropologie funéraire et épidémiologie. *Socio-anthropologie* **22,** 107–122 (2008).

64. Chaumoitre, C., Signoli, M., Dutour, O. & Panuel, M. Analyse des stries d'arrêt de croissance sur deux populations du XVIII ème siècle provenant de charniers de peste de Marseille et Martigues. in *LA PESTE entre épidémies sociétés.* (ed. Signoli M. , Chevé D. , Adalian P. , Boëtsch G. et Dutour O.) 83–89 (Erga, Firenze, 2007).

65. Signoli, M., Chausserie-Laprée, J. & Dutour, O. Etude anthropologique d'un charnier de la peste de 1720-1721 à Martigues. *Préhistoire Anthropol. méditerranéennes* **4,** 173–189 (1995).

66. Tzortzis, S. & Signoli, M. Les tranchées des Capucins de Ferrières (Martigues, Bouches-du-Rhône, France). Un charnier de l'épidémie de peste de 1720 à 1722 en Provence. *Comptes Rendus Palevol* **8,** 749–760 (2009).

67. Fiscella, G. N., Bennike, P. & Lynnerup, N. Transverse-" Harris"-Lines in a Skeletal Population from the 1711 Danish Plague Site. *Anthropologischer Anzeiger* **66**, 129–138 (2008).
